# Conservative Management of Emphysematous Gastritis With Gastric Mucosal Ischaemia: A Case Report

**DOI:** 10.7759/cureus.34656

**Published:** 2023-02-05

**Authors:** Marek A Bak, Ashray Rajagopalan, Geraldine Ooi, Mithra Sritharan

**Affiliations:** 1 Department of General Surgery, Monash Health, Melbourne, AUS; 2 Department of Upper Gastrointestinal and Hepatobiliary Surgery, Monash Health, Melbourne, AUS

**Keywords:** gastric ischaemia, gastric emphysema, gastritis, conservative management, exploratory laparotomy, upper endoscopy, emphysematous gastritis

## Abstract

Emphysematous gastritis is a rare condition historically associated with high mortality. It is characterised by gastric mural pneumatosis and portal venous gas, secondary to bacterial or fungal invasion. Given the rarity of the condition, there is little evidence to guide clinical decisions regarding whether a patient requires surgical resection. We describe the case of a 72-year-old male diagnosed with emphysematous gastritis, with endoscopic evidence of gastric fundus mucosal ischaemia. As there was no evidence of ischaemia extending to the serosa on exploratory laparotomy, gastrectomy was not performed, and the patient was managed conservatively. He subsequently made a full recovery, and was discharged without any further complications. This case demonstrates that in the absence of full-thickness gastric ischaemia, patients with emphysematous gastritis may be appropriate for conservative management without surgical resection.

## Introduction

Emphysematous gastritis is a rare and often lethal form of gastritis secondary to stomach wall invasion by gas-forming organisms. It was first described in 1889, characterised by gastric intramural gas and portal venous gas secondary to infection by gas-forming organisms [1-3]. These include Staphylococcus aureus, Streptococcus species, Escherichia Coli, Enterobacter species, Clostridium welchii, and rarely fungi such as Candida species [1].

Patients may present with nausea, vomiting, abdominal pain, distension, haematemesis and melaena, with a high prevalence of systemic toxicity [4]. Risk factors include those which predispose patients to infection, such as diabetes, and immunosuppression, as well as those which damage the gastric lining, such as alcohol abuse, ingestion of corrosive substances, or gastric ulcers [4-5]. Diagnosis is typically made on cross-sectional imaging. Features of emphysematous gastritis on computed tomography (CT) scans may include gastric wall thickening with intramural gas and portal venous gas [6-7]. In severe cases with full-thickness mural injury and perforation, free fluid or pneumoperitoneum may be seen [7].

Gastric emphysema without infection is a distinct clinical entity from emphysematous gastritis and is typically benign and self-limiting [7]. This may be precipitated by mechanical injury to the stomach wall, such as trauma from nasogastric tube placement, blunt trauma (e.g., cardiopulmonary resuscitation), or increased intraluminal pressure, such as in the setting of profuse vomiting, or gastric outlet obstruction [6]. Gastric emphysema alone seldom presents with acute abdomen, systemic toxicity or haemodynamic instability, and typically resolves spontaneously without the need for intervention [6,8].

Due to the rarity of the condition and absence of clear diagnostic criteria or treatment guidelines, management decisions are highly dependent on the clinical features of each patient, and the judgement of the treating clinician. Poor prognostic factors associated with increased mortality include elevated lactate (>2.0mmol/L) and haemodynamic instability [9]. These factors may influence treating clinicians to escalate to endoscopic or surgical evaluation to determine a need for gastric resection.

This case describes a patient with emphysematous gastritis, with evidence of gastric ischaemia on endoscopy, but with normal appearance of gastric serosa on laparotomy, who ultimately recovered without the need for gastrectomy.

## Case presentation

A 72-year-old Caucasian male presented with acute onset vomiting (without haematemesis), abdominal pain and distension two days after presentation with a thromboembolic stroke. He had received thrombolysis and endovascular clot retrieval. His past history was significant for myocardial infarction and coronary stenting, and atrial fibrillation (AF), treated with aspirin but no anticoagulation. He had no known history of heavy alcohol use.

He presented mildly tachycardic (heart rate 104, in atrial fibrillation), with other vital signs within normal limits. He had a distended abdomen and epigastric tenderness, without peritonism.

**Figure 1:**
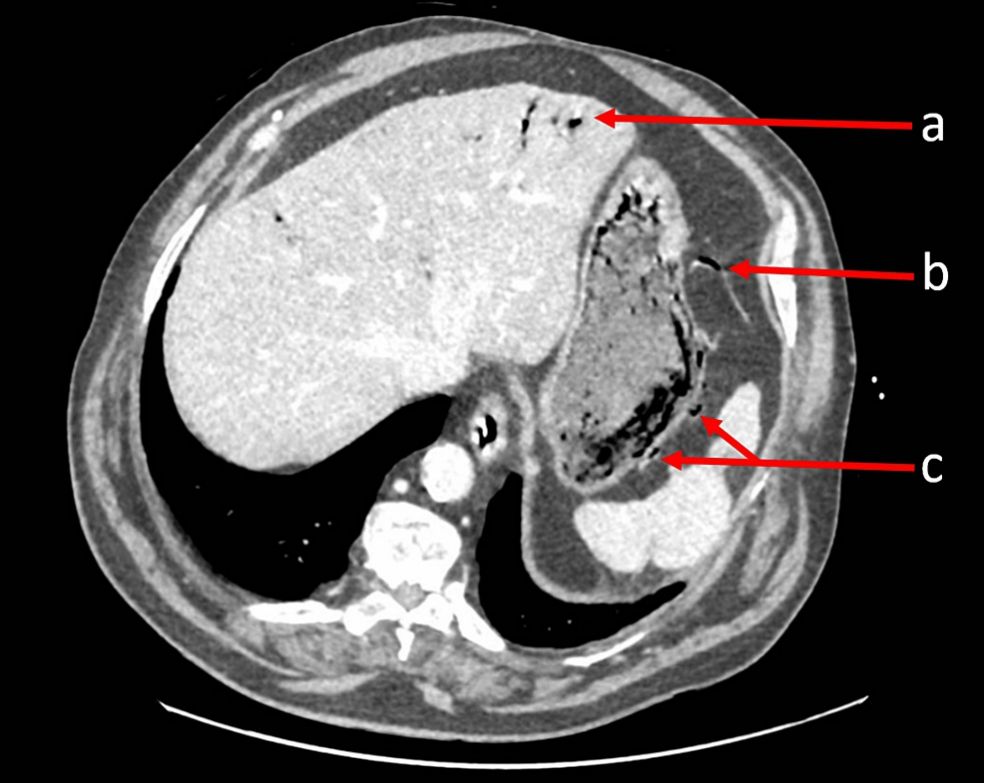
Axial portal-venous phase CT abdomen and pelvis (a) Hepatic portal venous gas. (b) Mesenteric venous gas. (c) Gastric intramural gas locules.

Portal venous phase-contrast CT of his abdomen revealed intramural gas within the stomach, and portal venous gas, without pneumoperitoneum (Figure 1). Serum lactate was elevated at 2.7 mmol/L (reference range: 0.5-2.0 mmol/L), as was his white cell count (WCC), at 12.6 x 10^9^/L (reference range: 4.0-11.0 x 10^9^/L).

The patient was made nil-by-mouth. A nasogastric tube was inserted for gastric decompression, with no bile or blood evident in the aspirated gastric contents. He was commenced on a high-dose intravenous proton-pump inhibitor (PPI).

**Figure 2:**
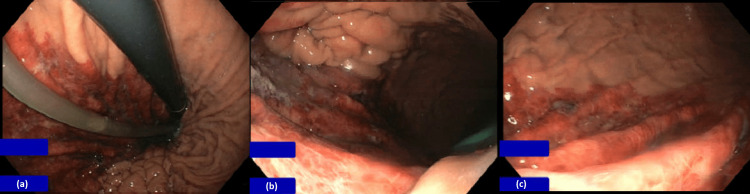
Gastroscopy showing the extent of gastric mucosal ischaemia (a) View of gastric cardia and fundus in retroflexion. (b) and (c) View of proximal body of the stomach.

Although the patient was haemodynamically stable and without features of acute abdomen, the treating surgeon made the decision to proceed with early endoscopic evaluation to identify a possible need for surgery before any potential haemodynamic deterioration. The acuity of the patient’s symptoms, coupled with the modest rise in lactate and WCC raised concerns of possible further deterioration with a working diagnosis of emphysematous gastritis. A differential of thromboembolic event was considered, given the patient’s presentation with acute stroke and background of AF without anticoagulation. Gastroscopy demonstrated mucosal ischaemia extending from the gastroesophageal junction to the gastric fundus (Figure 2), without perforation.

A decision was made to forgo laparoscopy and proceed directly to exploratory laparotomy, to provide adequate and safe exposure of the gastric fundus, as laparoscopic evaluation of the fundus would have required more manipulation of the stomach, increasing the risk of iatrogenic injury in the presence of ischaemia. Intraoperative findings noted a normal appearance of the gastric serosa, with no evidence of full-thickness ischaemia or gastric perforation. Other intraabdominal viscera appeared normal. Ultimately, no gastric resection was performed - this decision was driven by the absence of full-thickness injury and would likely not have changed even if the isolated mucosal injury was more extensive.

The patient was transferred to the Intensive Care Unit post operatively and had an uncomplicated recovery. Clear fluid intake was reintroduced on post-operative day 1, and the patient was transferred to the ward on day 2 with gradual upgrade of diet thereafter. He was discharged to stroke rehabilitation on post-operative day 10, with no post-operative complications noted during his admission.

## Discussion

Emphysematous gastritis presents a diagnostic and management challenge to clinicians, due to the rare nature of this condition and the absence of any specific diagnostic criteria or management guidelines. The symptoms which patients typically present with, such as abdominal pain, bloating, or vomiting, are fairly non-specific. Furthermore, clinical and imaging findings overlap with the significantly more benign diagnosis of gastric emphysema. As a result, decisions around whether to escalate to endoscopic or operative interventions can be difficult, as one must balance the risk of further deterioration due to a delay in treatment, against the risk of iatrogenic complications due to a potentially unnecessary procedure.

Current literature reveals a substantial reduction in mortality from emphysematous gastritis over time. Since the condition was first described, outcomes have improved significantly - Mortality rates have fallen from 61% pre-1990 to 33% post-2000 [2]. This corresponds with increased quality and accessibility of cross-sectional imaging, endoscopy, laparoscopy, and a decreased reliance on exploratory laparotomy [2]. Although countless other changes in medical practice over such a long period would make such a direct comparison difficult, it does suggest that more conservative management in selected patients may be appropriate.

Recently published case reports of patients with emphysematous gastritis suggest a trend towards more conservative management with broad-spectrum antibiotic cover, PPIs, gastric decompression, and bowel rest, with generally positive outcomes [7]. However, clinicians should be vigilant for evidence of more severe pathology and consider intervention when patients show evidence of systemic toxicity, ischaemia, or perforation - current literature shows that haemodynamic instability and raised serum lactate are both associated with increased mortality [9].

Our case demonstrates that the presence of gastric mucosal ischemia on endoscopy in the setting of emphysematous gastritis does not necessarily indicate full-thickness necrosis, and that in this setting, patients can be managed without gastric resection. The decision to proceed with diagnostic laparoscopy or laparotomy must be made at the discretion of the treating surgeon, considering each patient’s clinical presentation and risk of further deterioration.

## Conclusions

Emphysematous gastritis is a rare and potentially fatal condition. Patients often present with non-specific clinical features and diagnosis typically requires CT imaging. It shares many features with non-infective gastric emphysema, which is benign and self-limiting. Differentiating the two requires an understanding of patient risk factors or precipitants, as well as recognising that haemodynamic instability and systemic toxicity favour a diagnosis of emphysematous gastritis rather than gastric emphysema. Endoscopic evaluation can reveal mucosal ischaemia, though this does not strictly suggest full-thickness ischaemia (and therefore need for surgical resection). Where there are concerns that full-thickness ischaemia or perforation may be present, diagnostic laparoscopy or laparotomy may be necessary to assess for such pathology. Recent case reports published suggest that in stable patients without features of acute abdomen, conservative management with bowel rest, PPIs, broad-spectrum antimicrobials may be sufficient. Close clinical observation in appropriately selected patients may limit unnecessary operative interventions and iatrogenic complications.
